# Robot-Assisted Proprioceptive Training with Added Vibro-Tactile Feedback Enhances Somatosensory and Motor Performance

**DOI:** 10.1371/journal.pone.0164511

**Published:** 2016-10-11

**Authors:** Anna Vera Cuppone, Valentina Squeri, Marianna Semprini, Lorenzo Masia, Jürgen Konczak

**Affiliations:** 1 Motor Learning and Robotic Rehabilitation Laboratory, Department of Robotics, Brain and Cognitive Sciences, Istituto Italiano di Tecnologia, Genova, Italy; 2 Neural Computation Laboratory, Center for Neuroscience and Cognitive Systems, Istituto Italiano di Tecnologia, Rovereto, Italy; 3 School of Mechanical & Aerospace Engineering, Nanyang Technological University, Singapore, Singapore; 4 Human Sensorimotor Control Laboratory, School of Kinesiology and Center for Clinical Movement Science, University of Minnesota, Minneapolis, MN, United States of America; University of Chicago, UNITED STATES

## Abstract

This study examined the trainability of the proprioceptive sense and explored the relationship between proprioception and motor learning. With vision blocked, human learners had to perform goal-directed wrist movements relying solely on proprioceptive/haptic cues to reach several haptically specified targets. One group received additional somatosensory movement error feedback in form of vibro-tactile cues applied to the skin of the forearm. We used a haptic robotic device for the wrist and implemented a 3-day training regimen that required learners to make spatially precise goal-directed wrist reaching movements without vision. We assessed whether training improved the acuity of the wrist joint position sense. In addition, we checked if sensory learning generalized to the motor domain and improved spatial precision of wrist tracking movements that were not trained. The main findings of the study are: First, proprioceptive acuity of the wrist joint position sense improved after training for the group that received the combined proprioceptive/haptic and vibro-tactile feedback (VTF). Second, training had no impact on the spatial accuracy of the untrained tracking task. However, learners who had received VTF significantly reduced their reliance on haptic guidance feedback when performing the untrained motor task. That is, concurrent VTF was highly salient movement feedback and obviated the need for haptic feedback. Third, VTF can be also provided by the limb not involved in the task. Learners who received VTF to the contralateral limb equally benefitted. In conclusion, somatosensory training can significantly enhance proprioceptive acuity within days when learning is coupled with vibro-tactile sensory cues that provide feedback about movement errors. The observable sensory improvements in proprioception facilitates motor learning and such learning may generalize to the sensorimotor control of the untrained motor tasks. The implications of these findings for neurorehabilitation are discussed.

## Introduction

Proprioceptive information is essential for the control of movement. Its fundamental role for upper limb, postural and gait control becomes evident when examining the effects of sensory deafferentation on motor function. For example, despite intact motor pathways, patients with large fiber sensory neuropathy are unable to modulate force during grasping [1,2] and to coordinate even simple multi-joint movements [3]. They rely heavily on vision to control reaching and locomotion, but visual feedback only partially restores motor function [4,5]. Motor impairments associated with proprioceptive dysfunction have also been reported in numerous other neurological diseases such as Parkinson disease [6,7], focal dystonia [8], and stroke [9–13].

There is a growing interest in understanding the functional link between proprioception and motor control and its role in fostering neural plasticity through learning [14]. Increasing evidence indicates that learning related changes are bidirectional. That is, proprioceptive function may be enhanced after learning a motor task [15] or, vice versa, proprioceptive sensory training may improve motor performance [16]. With respect to enhancing the proprioceptive senses, the term *proprioceptive training* has been used to describe interventions that seek to improve proprioceptive function. It focuses on the use of somatosensory signals such as proprioceptive or tactile afferents in the absence of information from other modalities such as vision. In the context of rehabilitation its ultimate goal is to improve or restore sensorimotor function [17]. For the sake of brevity and consistency with existing literature we use the term *proprioceptive training* throughout this paper, but the reader should be mindful that such training is always a form of *proprioceptive-motor training*, because proprioception it is inherently linked to bodily movement. Unlike, for example, auditory training to improve pitch perception, proprioceptive training typically involves limb or bodily motion or postures.

With respect to improving impaired motor performance due to proprioceptive dysfunction, several approaches have been suggested to substitute or to augment residual proprioception through feedback from other sensory modalities in the hope that it would stabilize or improve motor function. Such sensory substitution has been provided through visual displays, the modulation of auditory pitch or by attaching mechanical vibrating stimulators to the skin [18]. There have been additional attempts to directly stimulate somatosensory afferents through neural interfaces using penetrating or surface electrodes [19] with the same aim of enhancing residual proprioceptive function.

The usefulness of tactile vibratory stimulation to enhance proprioception is particularly plausible, because proprioceptive and tactile afferents both terminate and share overlapping networks in the somatosensory cortex [20,21]. In this context it is important to consider the differences between vibro-tactile stimulation and vibro-tactile feedback (VTF). Experimenters have used vibro-tactile stimulation as an unspecific, somatosensory co-stimulation of proprioception. For example, vibratory stimulation of the distal wrist musculature has been used to promote stability of the proximal arm during reaching in hemiparetic patients [22]. Others have used vibratory stimulation as feedback to indicate error direction and magnitude related to an unwanted trunk sway [23,24]. Only in the latter case does mechanical vibration of the skin provide movement-relevant information. In a recent proof-of-concept study [25] we investigated, if VTF can augment proprioceptive sensory information. We tested two different groups of healthy subjects, one trained only with haptic feedback and one with haptic and vibro-tactile feedback and found that only the group receiving the multimodal feedback significantly improved the proprioceptive acuity indicating that VTF may enhance a proprioceptive training regimen.

Despite numerous claims that behavioral therapies focusing on proprioception are useful in treating motor impairments, reports on the efficacy of proprioceptive training vary widely [17]. At present, it is not even established to what extent such sensory training actually improves proprioceptive function in healthy populations. In addition, there is a paucity of data clearly indicating if and by how much such sensory improvements translate into motor improvements. Finally, little is known of how additional movement-relevant feedback provided as tactile feedback enhances this sensorimotor training process.

To address this knowledge gap, this study objectively quantified improvements in proprioceptive function due to proprioceptive training in a group of healthy young adults. We used a robotic device to employ a haptic proprioceptive training program that focused on joint position sense and required the learner to make spatially precise wrist movements. Training occurred without vision and required goal-directed movements to haptically specified targets. In a subgroup of participants proprioceptive training was augmented by additional vibro-tactile, movement-related feedback (VTF) on the forearm to determine, if such added feedback enhanced proprioception. Finally, to understand if the effectiveness of such vibro-tactile feedback is dependent on anatomical location, we applied vibro-tactile feedback either to the ipsilateral or contralateral forearm of the trained wrist.

## Materials and Methods

### Subjects

Twenty-eight right-handed young adults (13 males and 15 females; age: 25.7 ± 4.0 years) with no known neuromuscular disorders and naïve to the tasks participated in the experiment. The research conformed to the ethical standards laid down in the 1964 Declaration of Helsinki, which protects research subjects and was approved by the ethics committee of the ASL3 of Genova (Italy). Each subject signed a consent form conforming to these guidelines.

### Apparatus and experimental setup

**Fig 1:**
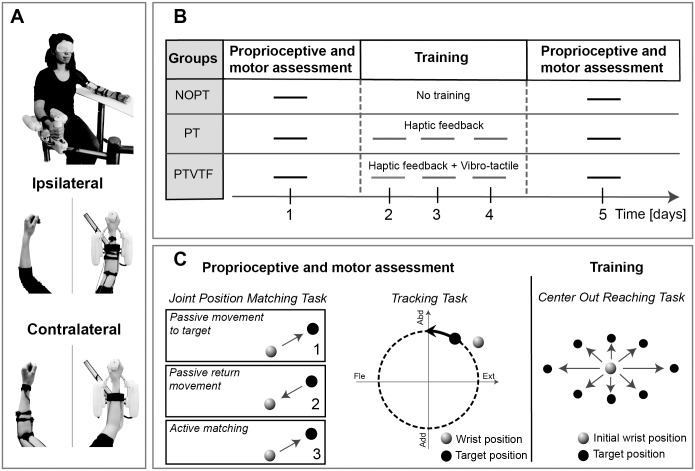
Experimental setup and training protocol. (**A**) The 3 degrees-of-freedom robot (WristBot) and the placement of the vibro-tactile actuators in the ipsilateral and contralateral configuration. The 4 vibro-tactile actuators were placed on the forearm to indicate right, left, up and down directions in order to correct movement trajectory. (**B-C**) Overall training protocol. All groups underwent proprioceptive and motor assessment which consisted of joint position sense testing and the wrist tracking task. Training executed by PT and PTVTF groups consisted of a center-out reaching task with haptic and vibro-tactile feedback.

The experimental setup (Fig 1A) utilized a three degree-of-freedom (DoF) wrist robotic exoskeleton (Wristbot) described in detail elsewhere [26]. In short, the robot allowed for the independent activation of wrist flexion-extension (± 70°), wrist abduction-adduction (± 40°) and forearm pronation-supination (± 57°) within a mechanical workspace that closely matched the physiological range of motion. The robot is powered by 4 brushless DC motors: two motors for abduction-adduction allowing for gravity compensation and one motor for each of the two remaining DoFs. An impedance control scheme was used to generate an assistive force field based on relative positions between the target and the end-effector, with a 1 kHz sampling frequency for haptic rendering. Vibro-tactile feedback was generated using an additional setup worn by the subjects and made by 4 vibration motors (307–100 Precision Microdrives): such wearable setup can be used to provide vibrational feedback through the ipsilateral or contralateral limb to the exercised wrist (Fig 1A). A real time workstation controlled both the robotic device and the vibro-motors, by means of analog and digital channels from a PCI acquisition card (Sensoray, model 626), while four digital counters read the end effector positions from the optical encoders embedded in the wrist haptic device. The software environment was implemented on Real-Time Windows Target^™^.

### Experimental design

A pretest-posttest intervention design was employed with three groups (Fig 1B). Subjects were randomly allocated to three different groups: a control group (N = 7) who received no proprioceptive training (NOPT); a treatment group (N = 7), who received proprioceptive training only (PT); a second treatment group (N = 14), who received proprioceptive training with additional vibro-tactile feedback (PTVTF). In the PTVTF group vibro-tactile feedback was applied either to the left (contralateral) or right (ipsilateral) arm with an equal number of subjects in each subgroup (PTVTF_left_: N = 7; PTVTF_right_: N = 7).

Training consisted of three 1-hour sessions spread over three days. During training subjects performed discrete, goal-directed reaching movements (center-out task). Before and after training, proprioceptive function was assessed for measuring position sense acuity [27]. In addition, wrist-hand motor performance was tested in an untrained, continuous movement tracking task: the purpose of examining motor performance in this task that was not explicitly trained and was sufficiently different from the trained center-out task, allowed to determine whether sensory training may successively generalize to the motor domain. An overview of the design is shown in Fig 1B–1C and procedure details are described below.

### Procedure

Subjects sat on a chair and were asked to grasp the handle connected to the robot end-effector and their forearm was constrained by straps to a rigid holder, in such a way that the biomechanical joints axes were closely aligned with the axes of rotation of the robot. Particular care was taken to maintain the angle of the elbow joint at approximately 90°.

#### Pre- posttest proprioceptive and motor assessment

Joint position sense assessment. We employed an established method to assess proprioceptive acuity [28] at the beginning and at the end of whole experiment. With vision occluded, participants had to match a previously experienced joint position by actively moving the wrist to this position performing an Ipsilateral matching task [27] (Fig 1C left panel). First, the subject’s hand was passively moved along a minimum jerk trajectory by the robot from the central position to a predetermined position and then repositioned to the start position at the center (center-out-center movement). Then, upon an acoustic cue the subject attempted to match the previous position by actively moving the wrist to the target position and then holding it while verbally indicating to the experimenter that this was the matched joint position. Upon verbal confirmation the WristBot moved the end effector back to the central position to end the trial. One training block included 24 trials (3 movement repetitions each to 8 target positions, see Fig 1C) involving a single joint or the combination of wrist flexion/extension and abduction/adduction, depending on the target position. The targets were spaced on an ellipse with semi-major and semi-minor axis corresponding to 40° of flexion/extension and 15° of abduction/adduction, respectively, in order to follow the biomechanical anisotropy of wrist between the two DoFs.

Motor performance in the untrained tracking task. This task aimed at evaluating if improvements due to the center-out training can be generalized to a different exercise, performed in the same workspace but involving different coordination of the two wrist DoF. The task consisted in tracking a moving target that drew a circumference according to the following trajectory:

$$\{\begin{array}{c} {\text{x}}_{\text{TG}}\left(\text{t}\right)=\text{Rcos}\left(\frac{2\pi \text{t}}{\text{T}}\right)\\ {\text{y}}_{\text{TG}}\left(\text{t}\right)=\text{Rsin}\left(\frac{2\pi \text{t}}{\text{T}}\right) \end{array},$$ (1)

where x_TG_ and y_TG_ were the moving target coordinates on the coordinated axes, R was the radius of the circle, equal to 15° (Fig 1C center panel). The target stopped as the Euclidean distance between target and end effector exceeded a 5° threshold and it restarted its motion once this distance became lower than this threshold. The complete round nominal duration was equal to the 10s in case the target moved without stopping. The starting point was positioned at 15° of extension and at 0° of adduction. Each block of movements consisted of 10 circular movements covered in two directions, i.e. 5 clockwise circles and 5 counterclockwise circles. Participants were blindfolded and the robot provided haptic feedback about the desired trajectory by delivering a torque that attracted the end effector through the target:

$$Torque_{i}=K\times \left|\theta_{EEi}-\theta_{TGi}\right|,$$ (2)

where *i* corresponds to either the flexion/extension (FE) or abduction/adduction (AA). *θ*_*EE*_ and *θ*_*TG*_ indicate the angular position of the end effector and target and *K* was the stiffness coefficient (Nm/rad). The *K* value was continuously modulated according to the distance between the target and the end effector in order to progressively and smoothly modulate the haptic feedback: its magnitude increased if the distance was larger than 5° and decreased when subjects succeeded in tracking the moving target. The modulation of *K* was regulated by a ramp with a slope of 0.1 Nm*s/rad.

#### Proprioceptive training: wrist reaching task

In absence of visual feedback, subjects performed discrete center-out reaching movements to 8 haptically specified targets (Fig 1C right panel). When a target was actively reached under different feedback conditions, as explained in the next section, an acoustic feedback was given and the robot passively moved the subject’s wrist back to the center of the workspace before initiation of the next. The targets were equally spaced on an ellipse with semi-major and semi-minor axis corresponding respectively to 40° of flexion/extension and 15° of abduction/adduction (i.e. same workspace of the Joint position sense assessment task). The single session was organized into 7 target sets. Each target set included 3 reaching movements for each of the 8 targets, with a total of 24 center-out movements. The order of presentation of the peripheral targets within a target set was randomized. The experimental training protocol consisted of 3 sessions, in 3 consecutive days for a total of 504 trials.

Haptic feedback to specify the target. Because vision was blocked, for the trained wrist reaching task the WristBot rendered a haptic force feedback about the direction of the target at the handle during a trial. For each trial the feedback was a constant torque ruled by the equation:

$$F_{i}=k\frac{\;\left(\theta_{EEi}-\theta_{TGi}\right)}{\left|\left(\theta_{EEi}-\theta_{TGi}\right)\right|},$$ (3)

where ***i*** corresponds to either the flexion/extension or abduction/adduction. *θ*_*EE*_ and *θ*_*TG*_ indicate the angular position of the end effector and target, respectively, and ***k*** was the magnitude of the force field: at the beginning of each trial, when the subject was positioned on the central target, the ***k*** value increased with a ramp profile and stopped to a constant value as the subject began to move (speed ≥ 0.4rad/s). This means that the torques in flexion/extension and in abduction/adduction were constant during each trial. They represented the smallest torques necessary to perceive the direction of the haptic target.

Vibro-tactile feedback. Vibro-tactile feedback was provided by 4 small, encapsulated vibration motors placed on the forearm region of dermatome C6 and secured with velcro and adhesive tape to the skin according the 4 different coordinated directions of movement (Fig 1A). Each vibrator provided feedback for movement in a specific direction for the two DOF of the wrist. The lateral vibrator turned on during wrist flexion, the medial during extension, while the anterior vibrator was active during adduction and the posterior during abduction. While performing the center-out task, the real-time system checked whether the participant deviated from the ideal straight line path towards the target by evaluating the lateral deviation (defined as the vector connecting the current position of the end-effector to its orthogonal projection on the ideal trajectory). Feedback about the extent of the deviation from the ideal path was encoded by vibration amplitude and frequency in three frequency bands (70, 80, 90 Hz), until the error was detected and therefore corrected by the subject. Both amplitude and frequency increased as the deviation from the ideal path increased (Table 1). This implies that for one single DOF motion only one vibrator was active at a time, while two motors could vibrate during motions involving the combination of two DOFs. Note that the vibro-tactile feedback did not alter the task dynamics. Prior to testing we verified that each subject could detect and differentiate between the three levels of vibration feedback.

**Table 1:** Settings for providing vibro-tactile feedback.

Vibration Frequency [Hz]	Vibration Amplitude [g]	Range of lateral deviation [deg]
70	0.9	1.5–3
80	1	3–5
90	1.1	5–7

### Measurements

#### Pre-posttest outcome measures

*Position sense measure*: We quantified a joint position *Matching Error* (ME) defined as the angular distance between the final position of the end effector and the target position:

$$ME=2\text{arcsin}\left(\sqrt{{\text{sin}}^{2}\left|\frac{\Delta \theta }{2}\right|+\text{cos}\theta_{1}\text{cos}\theta_{2}{\text{sin}}^{2}\left|\frac{\Delta ʎ}{2}\right|}\right),$$ (4)

where Δθ = Y_tg_—Y_ee_ and Δʎ = X_tg_—X_ee_.

Motor performance measures of the untrained tracking task. To assess subject performance during tracking task, we evaluated the *Tracking Error* (TE), computed as the mean value of the instantaneous euclidean distance between the target and the end effector. It is a measure of accuracy and therefore for an ideal errorless tracking corresponds to a zero value. We further evaluated the magnitude of the haptic force guidance experienced by the participant during the tracking task. We first computed the vectorial sum of *Torque*_*FE*_ and *Torque*_*AA*_ (see Eq 2). Then, we averaged the summed torque for each of the 10 circle movements and finally computed the mean across each movement to obtain a global measure of *haptic force feedback* (HF).

#### Measures to evaluate training of the wrist reaching task

To map progress during the 3-day training phases (reaching task) we recorded the wrist angular position for each DOF at a sampling frequency of 100 Hz for each trial. The respective time-series data were smoothed with a 4th order Savitzky-Golay filter (window size = 150 ms, equivalent to a cut-off frequency of 11 Hz) and then used to estimate the first two time derivatives (i.e., velocity and acceleration). Resultant speed end effector was computed as:

$$Speed=\sqrt{v_{FE}{}^{2}+v_{AA}{}^{2}},$$ (5)

where *v*_*FE*_ and *v*_*AA*_ are the velocity components of FE and AA wrist movement respectively.

To assess changes in motor and sensory performance during the 3-day training period the following variables were analyzed.

*Movement time*: The movement time (MT) was defined as the time between movement onset and end; movement onset was identified as the first time when speed exceeded a threshold of 0.04 rad/s; movement end represented the time that the End Effector-Target distance was smaller than 0.05 rad for at least 50 ms (i.e. had reached the target).

*Maximum lateral deviation*: The maximum lateral deviation (MLD) was defined as the highest value of the lateral deviation for a given joint angular trajectory. It was computed as the minimum instantaneous angular distance between the instantaneous wrist position and the ideal trajectory, which is the segment that directly links the center of the workspace with the target position.

Number of movement units. The Movement units (MU) represents the number of peaks in the speed profile between movement onset and end, computed as the number of local minima in the speed profile. It measures the degree of segmentation of the reaching motion and serves as an indirect indicator of the smoothness of the movement.

### Statistical analysis

For each variable we performed a repeated measures ANOVA with two factors: GROUP (3 different groups: **PT**, **PTVTF**, **NOPT**) and TIME (before/after training or early/late training phase) in order to understand the effects of training. When the analysis yielded a significant main or interaction effect (p < 0.05), we subsequently applied paired t-tests. For these tests, the significance level was adjusted using Bonferroni correction to account for multiple testing.

After applying a Bonferroni correction, the significant p-value was set to p = 0.05/6 = 0.0083 for determining possible between-group differences at baseline (pretest) and for pre-posttest comparisons within groups (3 groups: PT, PTVTF, NOPT), which resulted in 6 separate tests (3 between-group comparisons at baseline and 3 within-group comparisons to assess treatment effects). This significant level was applied to the pre-posttest outcome measures (i.e. *Matching Error*, *Tracking Error* and *Haptic force feedback*).

The significant p-value was set to p = 0.05/4 = 0.0125 for measures that evaluated training effects (i.e. *Movement time*, *Maximum lateral deviation* and *Number of movement units*) of the wrist reaching task (2 training groups: PT and PTVTF), which resulted in 2 between-group comparisons at baseline and 2 within-group comparisons).

## Results

### Measures of motor learning during proprioceptive training

**Fig 2:**
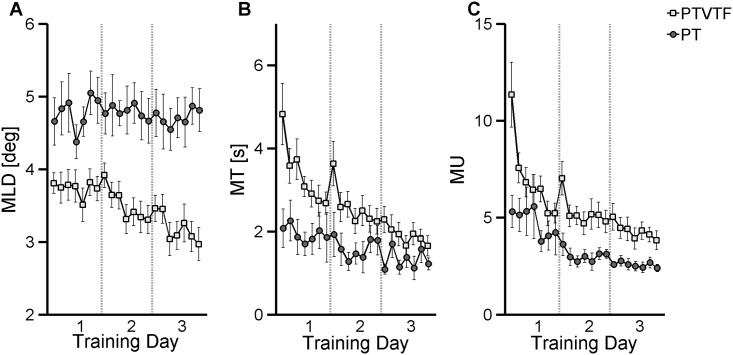
Motor learning related changes in the center-out pointing task during training. Each data point represents the group mean for a block of 24 trials (each training day consisted of 7 blocks; group mean = mean of individual subject means). Error bar represents the Standard Error. (**A**) Maximum Lateral Deviation (MLD). (**B**) Movement Time (MT). (**C**) Number of movement units (MU). **PT** = proprioceptive training; **PTVTF** = proprioceptive training + vibro-tactile feedback.

In order to evaluate the effect of motor learning during the 3-days of proprioceptive training on the two training groups (PT and PTVTF), we considered the change between early and late training (all values are reported as mean ± SE). As indicator of movement accuracy we evaluated the *maximum lateral deviation* (MLD) from the ideal straight-line trajectory. The effect of VTF on movement accuracy was evident with the PTVTF group exhibiting consistently lower MLD values throughout training (see Fig 2A).

Statistical analysis of MLD revealed a significant main effect for GROUP (F = 24, p < 0.001) and a significant GROUP x TIME interaction effect (F = 5.66, p = 0.02). The PTVTF group exhibited significantly lower *maximum lateral displacement* during the entire training period when compared to the PT group (Fig 2B; EARLY: PT vs PTVTF, p = 0.011). Yet, the PTVTF group significantly decreased MLD during training while the PT group did not (PTVTF: EARLY = 3.8° ± 0.14° vs LATE = 2.97° ± 0.23°, p = 0.0037; PT: EARLY = 4.65° ± 0.32° LATE = 4.81° ± 0.29°, p > 0.0125).

Other indicators of motor learning that were analyzed were *movement time* (MT) and *movement units* (MU). Fig 2B shows how MT changed for both training groups during the three days of training. Here the PT group revealed consistently faster performance than the PTVTF group during early training, but at the end of the training, both groups exhibited movement times that were no longer significant from each other. The respective ANOVA test yielded a significant TIME (F = 13.39 p = 0.0016), and GROUP effect (F = 6.75, p = 0.017) and a significant interaction between GROUP and TIME (F = 4.4 p = 0.049). The PTVTF significantly decreased MT with learning (EARLY 4.82 ± 0.74 s, LATE: 1.65 ± 0.24 s, p = 0.00078), while MT did not change significantly in the PT group (EARLY: 2.08 ± 0.46 s, LATE: 1.21 ± 0.14 s, p > 0.0125).

Training affected the smoothness of the executed movement as shown by training-related changes in movement units (Fig 2C). All groups significantly decreased the number of MU with training (TIME main factor: F = 15.91, p = 0.0008). The extent of decrease in MU was significantly different between groups (GROUP main factor: F = 8,73 p = 0.0082). During training the PT group mean MU decreased from 5.31 ± 0.83 to 2.39 ± 0.18 (EARLY vs LATE: p = 0.011), while PTVTF group mean decreased MU by 66%, from 11.34 ± 1.67 to 3.83 ± 0.49, (EARLY vs LATE: p = 0.001). The initial difficulty of the PTVTF group in performing a smooth movement was expressed by a higher MU value in early training (EARLY: PTVTF vs PT, p = 0.024 not significant after Bonferroni correction). The interaction between GROUP and TIME was not statistically significant (p > 0.05).

### Effect of proprioceptive training on joint position sense

**Fig 3:**
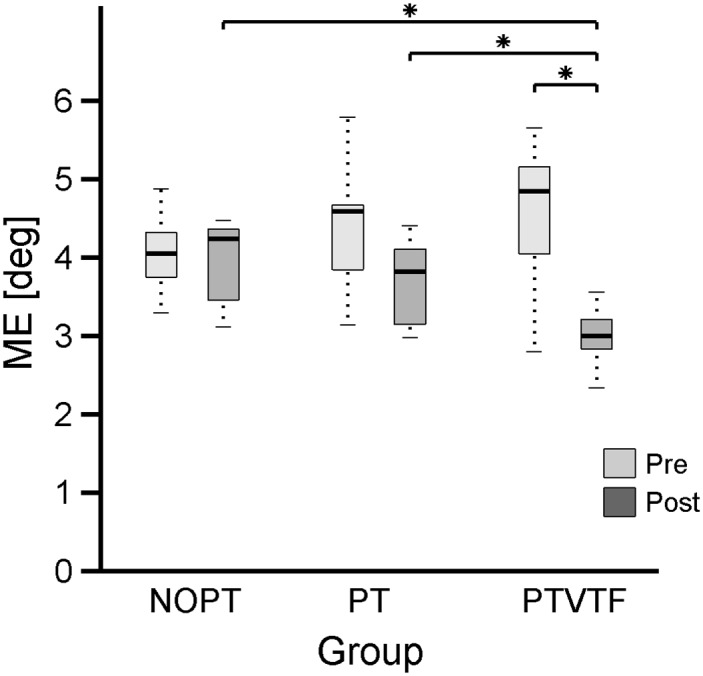
Effect of proprioceptive training on proprioceptive acuity. The boxplot shows the pre- and post-training distribution of the position matching error (ME) for each experimental group. Medians are indicated by the solid line inside the box. The lower and upper edges of the box represent the 25^th^ and 75^th^ percentiles, respectively. The whiskers indicate the 1^st^ and 99^th^ percentile. Asterisks (*) indicate statistically significant differences for between-group (POST: PT vs PTVTF; NOPT vs PTVTF) and within-group (PTVTF: PRE vs POST) comparisons.

We evaluated the effect of proprioceptive training in proprioceptive functions by comparing the pre and post training performance for joint position matching task. Analysis of the joint position matching error yielded a significant GROUP x TIME interaction effect (F = 5.7 p = 0.008). Subsequent within-group analysis revealed that the NOPT group mean for the *Matching Error* (ME) decreased by 3.44% (PRE: 4.06° ± 0.19°; POST: 3.92° ± 0.2°; p > 0.0083), while ME in the PT group decreased slightly by 17% (PRE: 4.37° ± 0.31; POST: 3.68° ± 0.2; p > 0.0083) and the PTVTF group mean ME decreased by 38% (PRE: 4.6°; POST: 3.05°; p = 0.00015). Between-group analysis showed that there were no significant differences of ME among groups prior to training (pre-test) confirming that our group data were unbiased (PRE: PT vs PTVTF, PTVTF vs NT, NT vs PT p’s > 0.0083). Fig 3 shows the relevant ME values for each group prior and post training.

### Generalization: Effect of proprioceptive training on motor function in the untrained motor task

To evaluate, if improvements in proprioceptive acuity may be generalized to improvements in motor precision, we analyzed the changes in movement errors and the use of haptic feedback in the tracking task that was not trained. We found that training had not led to a significant decrease in tracking error when the two proprioceptive training groups were compared to the between the NOPT group (PT, PTVTF; p’s > 0.05).

**Fig 4:**
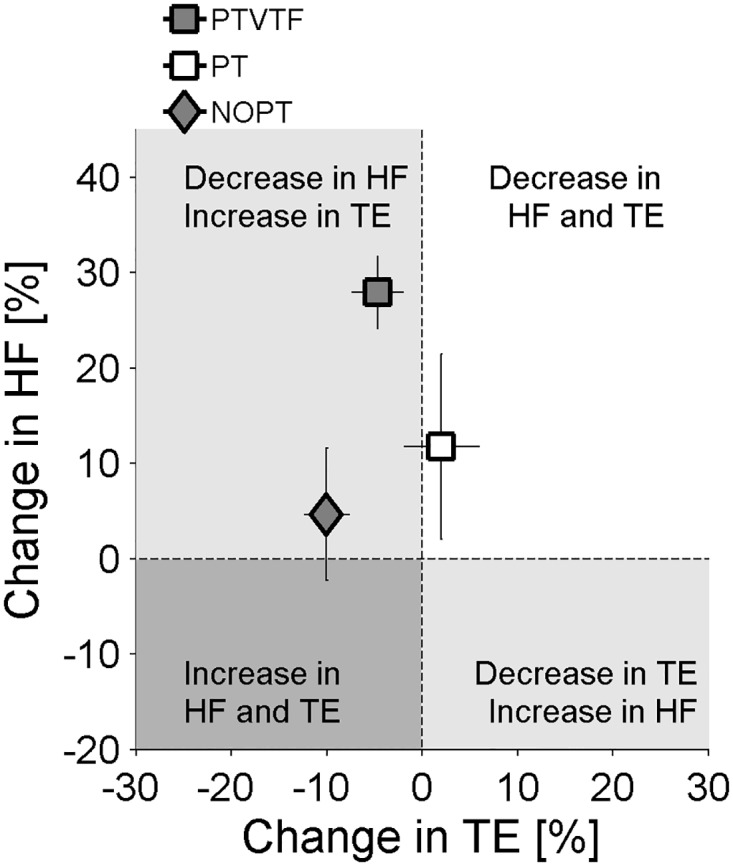
Effect of proprioceptive training on the untrained wrist tracking task. Pre to posttest change in the use of haptic force feedback (HF) as a function of tracking error (TE). Each data point represents the coordinates of the respective group means. The error bars represent Standard Error. The percentage of change was calculated as (initial value—final value) / initial value. Positive values correspond to a decrease of the variable with training. Note that the PTVTF group had significantly decreased the amount of haptic feedback after training.

In a second step we examined the use of haptic guidance as measured by the *haptic force feedback* (HF). The analysis of HF showed a significant effect for TIME x GROUP interaction (F = 3.85, p = 0.035). The only group that significantly decreased the amount of HF was the PTVTF group (p < 0.0001). The specific group values for TE and HF are shown in Table 2. Fig 4 shows the pre- to post-training change in TE as a function of HF illustrating that while the motor performance did not differ between groups their use of haptic feedback had changed after proprioceptive training.

**Table 2:** Tracking error and magnitude of haptic force in the untrained motor task.

Variables	Groups	PRE	POST	Statistic
Tracking error [deg]	NOPT	3.86 ± 0.14	4.21 ± 0.2	p>0.05
	PT	3.96 ± 0.2	3.79 ± 0.15	p>0.05
	PTVTF	3.88 ± 0.11	4.04 ± 0.1	p>0.05
Haptic force feedback [Nm]	NOPT	0.019 ± 0.002	0.018 ± 0.003	p>0.05
	PT	0.021 ± 0.002	0.017 ± 0.002	p>0.05
	PTVTF	0.021 ± 0.001	0.015 ± 0.001	P<0.0001

### Effect of vibro-tactile feedback location on proprioceptive and motor function

**Fig 5:**
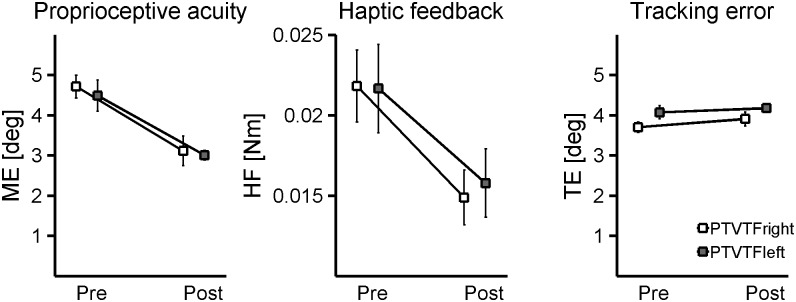
Effect of vibrotactile feedback location. Matching error (ME), haptic force feedback (HF) and tracking error (TE) values for PTVTF_right_ (light gray) and PTVTF_left_ (dark gray) groups. Each point represents group mean, pre and post training and the bars represent the SE. The comparison shows no difference between the two groups for each variable.

To investigate whether the location (ipsilateral or contralateral, Fig 1A) of the vibro-tactile feedback affected the proprioceptive and motor function, we applied the t-test comparison between PTVTF_right_ and PTVTF_left_ for pre- and post- training on all assessment variables. The analysis did not reach significance for any of the evaluated indicators between these two groups (p’s > 0.05). Fig 5 summarizes the relevant results for PTVTF_right_ and PTVTF_left_ for *matchinig error*, *haptic force feedback* and *tracking error*.

## Discussion

This study explored how a non-visual, haptic feedback-based sensorimotor training affects proprioception and motor learning. It compared a training that relied solely on proprioceptive and haptic guidance feedback with a learning paradigm that combined proprioceptive observational learning with vibro-tactile error feedback learning.

Specifically, we used a robotic device capable of providing haptic feedback and designed a training regimen that required the learner to make spatially precise wrist movements in the absence of vision. We assessed if proprioceptive training improved the acuity of the wrist joint position sense. Subsequently, we analyzed, if providing vibro-tactile movement feedback during training would enhance learning and be associated with better learning outcome measures. Finally, we checked if the spatial precision of wrist tracking movements, that were not trained, was improved.

The main findings of the study are the following: First, proprioceptive acuity of the wrist joint position sense was improved after proprioceptive training in the group that received vibro-tactile feedback. Second, the training had no impact on the spatial precision observed in the untrained wrist tracking task, but learners who received additional vibro-tactile movement feedback significantly reduced their reliance on haptic guidance feedback when performing the task. Third, the anatomical location for providing vibro-tactile feedback was not essential. In other words, VTF did not have to be applied to the trained limb. Learners who had received VTF to the contralateral limb equally benefitted from it. In the following, we will discuss our main findings and their implications in more detail.

### Sensory changes in proprioception due to training

We measured proprioceptive acuity of wrist joint position sense before and after training to determine to what extent proprioceptive training induces changes in the precision of the proprioceptive sense. Our results show that over the course of the 3-day training acuity of the wrist joint position sense did improve when a multimodal feedback was applied. However, the general conclusion that a sensorimotor training regimen focusing on proprioception enhances proprioceptive precision, needs to be qualified.

We found that the proprioceptive training group relying solely on haptic guidance (PT group) showed a slight, but statistically not significant improvement in proprioceptive acuity (+ 17%). In contrast, the group that received additional vibro-tactile feedback (PTVTF) exhibited a 38% increase in acuity (see Fig 3). Such magnitude of improvement in sensory acuity after a brief 3-day training is impressive and would be considered highly effective in a rehabilitation setting. From a neurophysiological perspective, two scenarios for the observed enhancements in proprioceptive acuity can be envisioned. First, VTF stimulates overlapping neuronal networks involved in the processing of somatosensory afferents such as in the somatosensory cortices [20]. Such co-stimulation could be unspecific to the motor task and it mainly serves to amplify neural activity in those regions that are central for forming proprioceptive percepts. Second, it is the movement related error feedback provided through VTF that is essential for inducing the observed changes in limb position sense. In other words, the sensory modality providing the relevant error cues is not important, but it is the saliency of the error information. We would contend that both factors plausibly play a role in enhancing proprioceptive function. With respect to the effect of sensory co-stimulation on movement perception, both visual-proprioceptive and tactile-proprioceptive co-stimulation has been shown to be highly effective in inducing kinaesthetic illusions of hand rotation [29]. However, in order to enhance the kinaesthetic illusion the bimodal sensory integration of tactile with proprioceptive stimuli need to be congruent [30].

When trying to understand the underlying neurophysiological processes responsible for the observed enhancements in limb position sense, it should be noted that our measure of proprioceptive acuity does not reflect exclusively the processing of afferent proprioceptive signals from the periphery. Because the applied joint matching method required an active joint movement to match the previously experienced joint position, it also reflects the processing of the integration of this *external* feedback with an *internal* feedback, or predicted sensory feedback, derived from the motor command [28,31–34]. That is, the described “sensory” effect may reflect changes in the processing of afferent feedback, as well as the updating of an internal forward dynamics model [35] and processes of integrating internal and external afferent feedback.

### Effects of proprioceptive learning on untrained motor function

Besides investigating to what extent the proprioceptive sense is trainable, we also examined, if such training affected motor performance. That is, could one find evidence that *proprioceptive training* as an intervention that seeks to improve proprioceptive function also improves sensorimotor function? This question is challenging to address experimentally. Unlike senses such as audition, where, for example, pitch perception can be trained in the absence of limb or body movement, proprioception necessarily requires movement. Thus, when evaluating the effectiveness of a somatosensory intervention to improve motor behavior, it is difficult to isolate the sensory from a motor aspect of training. In fact, one can argue that any form of motor learning is associated with proprioceptive processing and thus may train proprioception. In addition, there is evidence that such motor-sensory link is an integral part of sensorimotor learning [36,37]. Vice versa, one can argue that any form of proprioceptive training is *eo ipso* a form of motor training.

To address the issue of the embodiment of the proprioceptive sense, we had participants perform a second motor task that also required spatially precise wrist motion but was not part of the training. Because this task was not explicitly trained, one might assume that participants would not show meaningful improvements in the motor performance of this task. On the contrary, if participants did show signs of improved motor performance, then it was reasonable to conclude that such improvements could not simply be explained as “pure” effects of motor learning leading to improved motor control with neural changes primarily motor cortical areas involved in planning and execution. We therefore chose a continuous tracking task known to be sufficiently distinct from the discrete movement, goal-directed center-out task.

Our analysis revealed that proprioceptive training had no impact on the spatial movement precision in the untrained wrist tracking task. That is, the observable improvements in wrist proprioceptive acuity did not directly translate to a higher spatial precision of wrist movements, in general. In contrast to our result, a recent report revealed a direct motor effect as a function of somatosensory training [38]. In this study trajectory straightness was improved in a goal-directed reaching task after a 500-trial somatosensory training that required participants to sense the left-rightward deviation of a hand trajectory, while the hand was passively moved. Moreover, somatosensory training was associated with increases functional connectivity between anterior parietal brain regions (i.e. Brodmann area 2, bilateral primary somatosensory cortex), and dorsal frontal motor areas (i.e. left primary motor cortex, dorsal premotor cortex). That is, a network comprising somatosensory and motor areas showed elevated functional connectivity and this elevation was tightly related to the observed improvements in both motor and somatosensory domains.

In our view, the contradictory findings of the two studies relate to differences with respect to the “specificity of training” and may reflect the limitations of proprioceptive training. Both studies used discrete, goal-directed movements during somatosensory training. The motor transfer task in Vahdat et al. (2014) also involved short-duration discrete goal-directed movements, while in our experiment motor transfer required longer-duration continuous tracking movements. That is, the untrained motor task either involved similar types of hand trajectories (straight, with a defined start and end), and thus, sensory training had a high degree of specificity with respect to the motor transfer task, or it had lower specificity as for the continuous tracking task.

Both studies demanded the same amount of practice trials (500 vs. 504). It is therefore unlikely that differences in “dosage” can fully account for the differences between the two studies. In summary, a picture emerges from the current empirical evidence that shows that, in order to be effective, proprioceptive training may have to be closely tailored to the biomechanical constraints of the motor task. Somatosensory training may not easily generalize to a larger motor work- and task space.

### The role of somatosensory error feedback for improving motor control

Theories of sensorimotor learning typically distinguish between use-dependent learning, observational learning, reward-, and error-based learning [39]. Use-dependent learning implies that one learns by pure repetition, while observational learning is typically driven by providing visual information about a desired movement or movement target. In this study we did not provide a visual image of the desired trajectory or target but a haptic “image” of the trajectory. That is, our study participants observed haptically not visually. When those “pure” observational learners (PT group) were compared to those who also received somatosensory error feedback (PTVTF group), the trajectory kinematics during training revealed that observational learners in the PT group were faster and exhibited smoother trajectories in the early stages of learning. That is, during early learning the additional feedback modality actually degraded motor performance (see Fig 2B–2C). These initial differences in performance likely reflect that the VTF group had to learn how to use of the VTF to guide movement. That is, movement or proprioceptive-like feedback from a sensory modality typically not utilized for movement guidance needed to be incorporated in the underlying processes of sensorimotor integration necessary for trajectory control. In addition, the early superior performance of the observational learners of PT group in terms of movement time and movement smoothness began to vanish during the second training day. By the end of training on day 3, the PTVTF group generated joint trajectories that were similarly fast and smooth when compared to their PT group counterparts.

In contrast to the motor performance decrements observed in learners that have only haptic feedback available during early learning, VTF produced clear performance benefits from the beginning of training. Those learners who received VTF produced straighter trajectories already in the first training blocks indicating that they were able to incorporate this new spatial error feedback quickly and to use it throughout the 3-day training. In conclusion, the above findings underline the notion that the availability of haptic guidance feedback is not or only partially sufficient for the learning of the spatial characteristics of limb or end effector trajectories [40]. The availability of movement feedback from another somatosensory modality significantly aided trajectory formation leading to consistently straighter trajectories (see Fig 2A–2B). That is, the coupling haptic observational with somatosensory error feedback learning was beneficial for motor learning. These results are consistent with previous research on motor learning that found that multimodal is superior to unimodal learning and that augmented feedback from an additional modality promotes motor learning [41].

When considering the generalization or transfer of somatosensory-based training on untrained movements, we could not confirm that proprioceptive training had general effect on the motor domain such as improving spatial precision of trajectories. However, we did observe a generalized “sensory” effect. Learners who received additional vibro-tactile movement feedback during training, significantly reduced their reliance on haptic guidance feedback when performing the untrained tracking task.

Finally, we found that the augmented feedback did not necessarily have to be provided to the trained limb segment directly. That is, learning did not depend on providing vibro-tactile feedback to the trained wrist. Learners who had received VTF to the contralateral limb equally benefitted from it. Neither differences in motor measures (*tracking error*), nor sensory measures (magnitude of *haptic feedback* or proprioceptive acuity expressed by the *matching error*) were observed when applying VTF to the forearm of the trained wrist joint or to the contralateral forearm (see Fig 5). This implies that the applied movement trajectory feedback from another somatosensory modality was salient during learning. However, it was not relevant where the feedback was applied. It is possible that the underlying neural pathways that could facilitate such transfer of somatosensory-based movement feedback are transcallosal projections from the primary or secondary somatosensory cortices to homotopic and heterotopic regions in the contralateral cortex [42], which, in turn, are known to project to motor cortical areas [43]. In other words, VTF originating from the homologous region of the untrained arm could reach motor cortical regions of the trained arm via projections crossing the corpus callosum connecting the two cortical hemispheres.

This finding does have relevance to treatment regimen designed to retrain motor function in people with somatosensory deficits due to neurological disease such as cortical stroke, because it implies that training effects can be achieved by providing somatosensory feedback to a less affected limb.

### Conclusion

This study examined the trainability of the proprioceptive sense and explored the relationship between proprioception and motor learning. It showed that proprioceptive acuity can be enhanced significantly even with brief 3-day training and that such sensory change facilitates motor learning. Its results document the effectiveness of proprioceptive training especially when learning is coupled with tactile sensory cues that provide feedback about movement errors. Given that restoring somatosensory and sensorimotor function is a main goal of proprioceptive training when applied during rehabilitation (Aman et al. 2015), our results are promising. They add to the increasing evidence that proprioceptive training enhances somatosensory and motor learning (e.g. Wong et al. 2011, 2012; Vahdat et al. 2014) may become an effective behavioral intervention for treating movement disorders.

## Supporting Information

S1 Table Assessment Variables. Values of the three assessment variables (Matching Error, Haptic Feedback and Tracking Error) for each single subject in two phases: Pre training and Post Training.
(PDF)

S2 Table Training Variables. Values of the three training variables (Max Displacement, Movement Time and Movement Units) for each single subject in two phases: Early training and Late Training.
(PDF)
