# Supplementary material for: Robot-Assisted Proprioceptive Training with Added Vibro-Tactile Feedback Enhances Somatosensory and Motor Performance
Source: PLoS One. 2016 Oct 11;11(10):e0164511. doi: 10.1371/journal.pone.0164511 (PMC5058482; doi:10.1371/journal.pone.0164511)
Supplement: S1 Table — Values of the three assessment variables (Matching Error, Haptic Feedback and Tracking Error) for each single subject in two phases: Pre training and Post Training. (PDF) [file pone.0164511.s001.pdf]

S1 Table. Assessment Variables. Values of the three assessment variables (Matching Error, Haptic Feedback and Tracking Error) for each single subject in two phases: Pre training and Post Training.

Matching Error [deg]			
**Groups**		**TIME**	
NOPT		PRE	POST
	subj1	4,037325542	4,390700311
	subj2	4,222607373	3,398613411
	subj3	4,867372666	4,232426865
	subj4	3,650322359	4,468002706
	subj5	4,045806987	3,116858784
	subj6	4,3525201	4,266748801
	subj7	3,289141128	3,615496563
	MEAN±SE	4,0664±0,19	3,927±0,2
PT			
	subj1	4,662333759	3,439482831
	subj2	4,001108605	3,89998786
	subj3	4,669899772	4,405079045
	subj4	4,583764101	2,977509877
	subj5	3,136690096	3,046356749
	subj6	3,790072457	3,81507411
	subj7	5,779954873	4,174182676
	MEAN±SE	4,3748 ±0,32	3,6797±0,2
PTVTF			
PTVTFright	subj1	4,50058613	2,336017134
	subj2	5,001670751	2,050313611
	subj3	3,258464015	3,556495033
	subj4	5,414464768	2,835120512
	subj5	4,678098566	4,990232833
	subj6	5,481393908	2,848038771
	subj7	4,627267154	3,138165126
PTVTFleft	subj8	2,794705509	2,403280365
	subj9	3,616507603	2,874065899
	subj10	4,04629464	3,187204876
	subj11	5,649242817	2,96384747
	subj12	5,011555825	3,211832177
	subj13	5,155057601	3,32620632
	subj14	5,122341507	3,035746565
	MEAN±SE	4,597 ±0,23	3,054±0,18

Haptic Feedback [Nm]			
**Groups**		**TIME**	
NOPT		PRE	POST
	subj1	0,026010067	0,030160443
	subj2	0,015099919	0,014667181
	subj3	0,022422705	0,02414724
	subj4	0,017102957	0,011694713
	subj5	0,019330243	0,01664568
	subj6	0,012531053	0,012433142
	MEAN±SE	0,01875±0,002	0,018291±0,003
PT			
	subj1	0,018578801	0,014327521
	subj2	0,029734237	0,024298708
	subj3	0,022578744	0,022664995
	subj4	0,022847786	0,009830299
	subj5	0,011924999	0,014688369
	subj6	0,021698784	0,024031905
	subj7	0,021232672	0,017977434
	MEAN±SE	0,021228±0,002	0,01826±0,002
PTVTF			
PTVTFright	subj1	0,017614587	0,010151519
	subj2	0,023629938	0,01558902
	subj3	0,019268121	0,017482635
	subj4	0,011949863	0,008122108
	subj5	0,028041483	0,014805083
	subj6	0,02373121	0,021568796
	subj7	0,028412384	0,016385963
PTVTFleft	subj8	0,01817601	0,015154527
	subj9	0,022781793	0,012500163
	subj10	0,027158389	0,020617811
	subj11	0,011486961	0,008009111
	subj12	0,030776474	0,018556238
	subj13	0,013998639	0,011486411
	subj14	0,027148396	0,02402806
	MEAN±SE	0,02172±0,0017	0,015318±0,00131

Tracking Error [deg]			
**Groups**		**TIME**	
NOPT		PRE	POST
	subj1	3,360482196	3,575038411
	subj2	3,83941684	4,500760322
	subj3	3,761853215	3,847875647
	subj4	4,102833314	4,777716675
	subj5	3,726746052	3,957520272
	subj6	4,355215752	4,622779361
	MEAN±SE	3,8577±0,139	4,2136±0,198
PT			
	subj1	4,817689946	4,144272587
	subj2	3,811179597	3,819835995
	subj3	3,394258712	3,132340064
	subj4	3,621353926	4,199432619
	subj5	4,460139746	4,032217711
	subj6	3,917432831	3,410070274
	subj7	3,716675954	3,81349912
	MEAN±SE	3,962676±0,189	3,7931±0,1488
PTVTF			
PTVTFright	subj1	3,970962686	3,652741769
	subj2	3,635740074	3,547846991
	subj3	4,017571702	3,847325937
	subj4	4,127132092	4,871394274
	subj5	3,388204689	3,988311245
	subj6	3,449413134	3,888426807
	subj7	3,302359514	3,512940858
PTVTFleft	subj8	4,402082619	4,547421724
	subj9	3,773378031	4,072772248
	subj10	3,933427764	4,082374954
	subj11	4,840225582	4,338083511
	subj12	3,610733736	4,088163691
	subj13	4,111473663	4,310220513
	subj14	3,804173654	3,759966028
	MEAN±SE	3,88335±0,11	4,036±0,1029
