# Supplementary material for: Robot-Assisted Proprioceptive Training with Added Vibro-Tactile Feedback Enhances Somatosensory and Motor Performance
Source: PLoS One. 2016 Oct 11;11(10):e0164511. doi: 10.1371/journal.pone.0164511 (PMC5058482; doi:10.1371/journal.pone.0164511)
Supplement: S2 Table — Values of the three training variables (Max Displacement, Movement Time and Movement Units) for each single subject in two phases: Early training and Late Training. (PDF) [file pone.0164511.s002.pdf]

S2 Table. Training Variables. Values of the three training variables (Max Displacement, Movement Time and Movement Units) for each single subject in two phases: Early training and Late Training.

**Max Displacement [deg]**			
**Groups**		**TIME**	
PT		Early	Late
	subj1	3,95636417	4,929480501
	subj2	5,555281511	5,707835263
	subj3	5,969355341	5,568132502
	subj4	4,722622781	4,070607613
	subj5	3,873539806	5,248393862
	subj6	3,766516012	4,537258574
	subj7	4,740251153	3,612843727
	MEAN±SD	4,6548±0,325	4,81065±0,29446
PTVTF			
PTVTFright	subj1	3,389799137	3,450475025
	subj2	2,882981009	2,206259059
	subj3	3,685837316	2,71233067
	subj4	4,214944767	4,42578675
	subj5	3,991672794	4,522552718
	subj6	3,602785179	4,061109444
	subj7	3,454322697	2,291368753
PTVTFleft	subj8	3,815656777	2,288557159
	subj9	4,113729805	2,662062593
	subj10	3,709476876	2,385519834
	subj11	4,580647358	2,102332402
	subj12	4,564254727	3,413554737
	subj13	4,305488338	2,59431438
	subj14	2,980162605	2,436115136
	MEAN±SD	3,806554±0,141	2,968±0,22651

**Movement Time [s]**			
**Groups**		**TIME**	
PT		Early	Late
	subj1	1,699375	0,907083333
	subj2	1,950666667	1,6325
	subj3	3,535714286	1,54625
	subj4	4,022666667	0,66875
	subj5	0,995625	1,299166667
	subj6	1,280714286	1,57625
	subj7	1,055384615	0,88875

	MEAN±SD	2,07716±0,461	1,2169±0,148
PTVTF			
PTVTFright	subj1	3,436923077	1,101666667
	subj2	3,136	1,648333333
	subj3	4,246875	3,72375
	subj4	3,031428571	1,497916667
	subj5	1,819285714	0,982916667
	subj6	4,620714286	1,40625
	subj7	8,551428571	2,478333333
PTVTFleft	subj8	2,865833333	0,962083333
	subj9	5,461428571	1,467083333
	subj10	3,428461538	2,864583333
	subj11	0,919333333	0,775
	subj12	9,401428571	1,33125
	subj13	9,264666667	2,42125
	subj14	7,345714286	0,460833333
	MEAN±SD	4,82353±0,74	1,6515±0,242

**Movement Units**			
**Groups**		**TIME**	
PT		Early	Late
	subj1	3,8125	2,166666667
	subj2	7	2,833333333
	subj3	7,357142857	3,166666667
	subj4	8,2	1,875
	subj5	2,4375	2,333333333
	subj6	3,6	2,5
	subj7	4,785714286	1,875
	MEAN±SD	5,31326±0,832	2,39286±0,1823
PTVTF			
PTVTFright	subj1	6,5	2,333333333
	subj2	10,5	3,375
	subj3	9,1875	7,416666667
	subj4	7,4	4
	subj5	5,066666667	2,333333333
	subj6	8,214285714	3,5
	subj7	15,5	5,291666667
PTVTFleft	subj8	12,42857143	2,958333333
	subj9	11	3,625
	subj10	8,4	7,041666667

	subj11	2,333333333	2,166666667
	subj12	25,28571429	2,708333333
	subj13	15,86666667	5,5
	subj14	21,06666667	1,375
	MEAN±SD	11,33924±1,676	3,8303±0,49
